# Antibacterial effect of different concentrations of sodium hypochlorite on *Enterococcus faecalis* biofilms in root canals

**DOI:** 10.15171/joddd.2017.038

**Published:** 2017-12-13

**Authors:** Mohammad Forough Reyhani, Yousef Rezagholizadeh, Mohammad Reza Narimani, Lotfollah Rezagholizadeh, Mohammad Mazani, Mohammad Hossein Soroush Barhaghi, Yavar Mahmoodzadeh

**Affiliations:** ^1^Department of Endodontics, Faculty of Dentistry, Tabriz University of Medical Sciences, Tabriz, Iran; ^2^Iranian Center of Excellence in Health Management, Tabriz University of Medical Sciences, Tabriz, Iran; ^3^Department of Biochemistry, School of Medicine, Ardabil University of Medical Sciences, Ardabil, Iran; ^4^Department of Microbiology, Faculty of Medicine, Tabriz University of Medical Sciences, Tabriz, Iran

**Keywords:** Antibacterial, biofilm, *Enterococcus faecalis*, sodium hypochlorite

## Abstract

***Background.*** The aim of this study was to evaluate the effectiveness of different concentrations of sodium hypochlorite (NaOCl) solution in reducing bacterial growth in Enterococcus faecalis biofilms in root canals.

***Methods.*** The root canals of maxillary central incisors of 104 subjects underwent chemomechanical debridement. In order to remove the smear layer, 5.25% sodium hypochlorite solution was used for 3 minutes in the root canals. Then, the samples were immersed in 1 mL of 17% EDTA for 3 minutes. Finally, the root canals were irrigated with phosphate-buffered saline (PBS) solution. After removing the smear layer, the samples were sterilized. Then E. faecalis biofilms formed within the root canals at 4-, 6-, and 10-week intervals were evaluated. Each group was divided into 4 subgroups in terms of the antibacterial treatment: group 1: 1% NaOCl solution; group 2: 2.5% NaOCl solution; group 3: 5.25% NaOCl solution; and group 4: PBS solution. After preparation of root canal filings, the counts of live bacteria were calculated through the classic method of counting, i.e. colony forming units (CFU), followed by the analysis of data.

***Results.*** In groups 2 and 3, there was no bacterial growth due to complete removal of E. faecalis biofilms (P<0001), while the bacterial counts in group 1 at 4-, 6- and 10-week intervals decreased compared to the control group.

***Conclusion.*** The bacterial cells in mature and old biofilms have higher resistance to 1% NaOCl solution compared to the young biofilms. However, the 2.5% and 5.25% NaOCl solutions caused complete inhibition of the growth of E. faecalis biofilm in all the stages of development.

## Introduction

Removal of bacteria from the root canal(s) and prevention of infection by microorganisms in the pulp and periapical tissues are the goals of endodontic therapy.^1^ Chemomechanical debridement of the root canal, which includes mechanical instrumentation and application of chemical cleaning, is the main process during elimination of canal bacteria.^1,2^ However, resistant strains of bacteria can survive in the root canal and cause root canal infection in spite of appropriate mechanical and chemical cleaning.^3^ While antibacterial agents have easy access to the planktonic bacterial cells in the root canal, bacteria in the biofilms adhering to the canal wall or located in the complex parts of the root canal such as the end portions of the dentinal tubules and lateral canals are less accessible to certain treatments; therefore, specific treatment strategies are needed to overcome such limitations.^4^

*E. faecalis* biofilm is a dynamic structure of bacterial populations enclosed in a polymeric polysaccharide matrix.^5^ This cohesive structure resists against antibacterial agents through three main mechanisms:^6^ 1) The thick and solid structure of biofilm does not allow antimicrobial agents to penetrate and access microorganisms; 2) Microorganisms in deeper layers of biofilms, under the influence of a concentrated gradient, are kept away from food sources, resulting in slow-growing cells that are more resistant; 3) Over time, the cells in the biofilm structure undergo phenotypic changes as a result of physiological and metabolic conditions in the biofilm environment which consequently lead to the proliferation of resistant phenotypes.

*E. faecalis* is the most common bacterial species isolated from failed root canals and periradicular infections. ^7^ This microorganism is an anaerobic grampositive coccus that can tolerate harsh environmental conditions, including high alkaline pH (such as calcium hydroxide), dry climate, and high concentration of salts. The ability of *E. faecalis* to penetrate into the dentinal tubules enables it to be safe from cleaning solutions and endodontic instruments. Furthermore, its ability to form biofilms in root canals plays an important role in its resistance against antimicrobial agents.^8^

NaOCl is the most effective root canal irrigation solution and a powerful disinfectant that has favorable characteristics such as tissue solubility and proteolytic as well as bactericidal effects on microorganisms and bacterial endodontic biofilms.^9^ The most common concentrations of NaOCl used as a root canal detergent in endodontic treatments are in the range of 0.5‒5.25%. With the increasing growth of *E. faecalis* biofilm, the biofilm structure becomes calcified and, as a consequence, the removal of this mature and mineralized biofilm through conventional methods becomes more difficult, ultimately leading to resistant root canal infections.^10^ It has been observed that signs of mineralization and full maturity appear after six weeks in *E. faecalis* biofilm. Therefore, the six-week period of its growth is considered as the maturity index of biofilms. Most of the previous studies were conducted on young biofilms, whereas in reality, most of the biofilms in root canals at the time of treatment are several weeks or months old.^11-13^ Therefore, the results of laboratory studies have no exact matches with clinical reality. Thus, studying the different stages of biofilm might be helpful in understanding the relationship between the organization and maturation of biofilm and its sensitivity to antimicrobial therapy.

The aim of present study was to investigate the bactericidal effects of different concentrations of NaOCl on biofilms of *E. faecalis* in the 3 stages of immature biofilm (4-week-old biofilm), mature biofilm (6-week-old biofilm), and old biofilm (10-week-old biofilm).

## Methods

This study was approved by the Ethics Review Committee for Research, Tabriz University of Medical Sciences, Tabriz, Iran. A total of 96 samples were examined in this study. The samples included 96 human maxillary central incisors with straight and mature roots which did not have root caries, had not undergone endodontic treatment and had been extracted for periodontal reasons. The presence of one canal in the root was confirmed by two periapical radiographs taken from mesiodistal and buccolingual directions.

Dental samples were kept in a solution of 0.5% chloramine T until used for the study. Any remnants of calculus and periodontal tissue were removed by an ultrasonic instrument (Cavitron, Dentsply Ltd Weybridge, UK). Then all the samples were cut by hard-coated diamonds (D&Z, Diamant, Germany) from near the cementoenamel junction (CEJ) so that only 12 mm of root length remained. Working lengths of all canals were measured using #15 K-file (Dentsply Maillefer, Ballaigues, Switzerland). First, the coronal two-thirds of the canals were prepared by #4 to #6 Gates-Glidden drills (Dentsply Maillefer, Ballaigues, Switzerland). Then, using the step-back technique, the root canals were instrumented up to #60 K-file. The root canals were irrigated with normal saline using a 2-mL syringe and a 30-gauge needle. After instrumentation, 5.25% NaOCl (Taj Corp, Tehran, IR) was injected into the canals for 3 minutes. Then, each sample was immersed in 1 mL of 17% EDTA (Pulpdent Corp, MA, US) for 3 minutes. Finally, the root canals were irrigated with phosphate-buffered saline (PBS) solution. After completing the cleaning process, three teeth were randomly selected and sectioned parallel to the root long axis. They were cut with a diamond disc near the canals; then their roots were bisected by a chisel. Cutting was carried out with a chisel in order to prevent accumulation of dentin chips on the root canal walls. Then the dentinal tubules were investigated under scanning electron microscope (SEM) (VEGAS Tescan, Cranberry, PA) to analyze the opening of the tubules and remove the smear layer.

The samples were autoclaved at 121°C and a pressure of 15 psi for 20 minutes. To verify the effectiveness of sterilization processes, all the samples were immediately placed in brain-heart infusion broth (Merck, Darmstadt, Germany) at 37°C for 24 hours. To create experimental biofilms, first a pure culture of microorganisms was prepared. To achieve a pure culture, the bacteria were incubated 37 °C in the presence of 1% CO2 for 24 hours; then the resultant culture was centrifuged to make a suspension. Microbial quality was confirmed using a visible UV spectrophotometer in terms of 0.5 McFarland standard. The standard suspension of *E. faecalis* was injected into each root canal and then every root was placed in a sterile tube. In order to keep the culture conditions stabilized and provide nutritional support, fresh food was injected into the canals every other day. During incubation, the temperature was kept constant at 37°C. After 4 weeks of *E. faecalis* biofilm growth, in order to perform 4-week biofilm processes, 32 roots were randomly selected and classified into 4 groups (n=8) in terms of the type of treatment in 4, 6 and 10 weeks:

Group 2: 2.5% NaOCl; The root canals were irrigated with 10 mL of 2.5% NaOCl for 10 minutes using a 10-mL plastic syringe and a 30-gauge needle.
Group 3: 5.25% NaOCl; The root canals were irrigated with 10 mL of 5.25% NaOCl for 10 minutes using a 10-mL plastic syringe and a 30-gauge needle.
Group 4: PBS; The root canals were irrigated with 10 mL of PBS for 10 minutes using a 10-mL plastic syringe and a 30-gauge needle.

All the root canals were dried with paper points after 10 minutes and irrigated again with normal saline solution. The samples were kept at -25°C for 24 hours. This low temperature (pre-cooling) prevents the killing of *E. faecalis* by the heat produced as a result of drilling samples. Then, a thin layer of root canal walls (dentin filings) were removed by #5 and #6 Gates-Glidden drills. The root canal dentin filings were weighed with a sensitive electronic weighing machine (Merck, Darmstadt, Germany) to obtain 10 mg of each sample.

Then dentin filings were placed in sterile tubes; 2 mL of normal saline was added to each tube and mixed for 20 seconds. After that, 10-fold serial dilutions were prepared up to the concentrations of 10^-7^. A total of 100 mL of each dilution was added to three Mueller-Hinton І agar (Merck, Darmstadt, Germany) plates and incubated at 37°C for 48 hours. All the procedures were performed inside a laminar flow chamber with sterile equipment. Classical techniques were used for counting *E. faecalis* bacteria on agar plates. Bacterial growth on agar plates with concentrations of 10^-7^, 10^-6^, and 10^-5^ were not considered. The average bacterial count (CFU) for 3 plates with concentrations of 10^-2^, 10^-3^, and 10^-4^ were estimated. Then the average was added to the concentration of 10^-3^ to obtain one CFU value for all the samples.

After 6 weeks of biofilm growth, 32 roots and after 10 weeks, 32 other roots were selected and randomly classified into 4 groups (n=8) based on the type of treatment they received in 4 ,6 and 10 weeks. All the processes mentioned for the 4 week-old biofilm were repeated on 6 week- and 10 week-old biofilms. Three teeth from each biofilm age groups were randomly selected and sectioned parallel to root long axis using a diamond disc in order to study the formation of young, mature and old biofilms using SEM.

Data analysis was performed using SPSS 22. Kolmogorov-Smirnov test was used to determine the normality of data. Based on the results of this test, data distribution was not normal. Thus, the non-parametric tests of Kruskal-Wallis and Mann-Whitney U were employed to study the differences between the groups. ANOVA was also used to compare the means of the groups.

## Results

**Figure 1:**
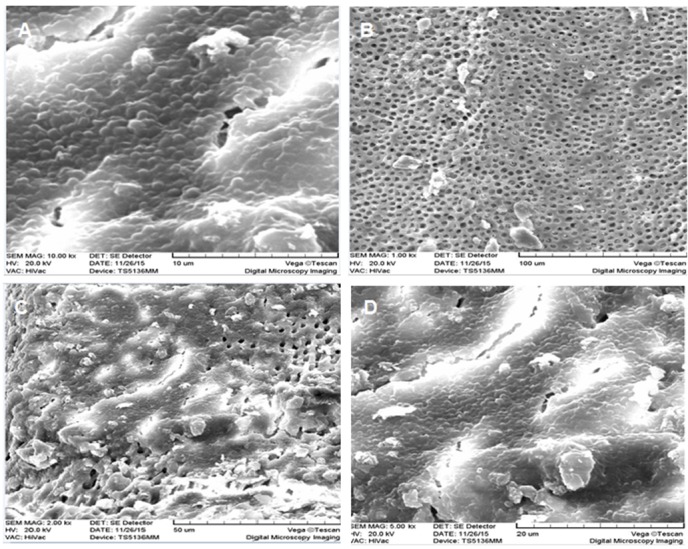
SEM analysis of the development of *E. faecalis* biofilm on the surface of root canal dentin. A: Overview of opening of dentinal tubules after removal of the smear layer and before incubation; B: *E. faecalis* biofilm topography after 4 weeks of incubation; C: *E. faecalis* biofilm topography after 6 weeks of incubation; D: *E. faecalis* biofilm topography after 10 weeks of incubation.

*E. faecalis* biofilms formed on the root canal surface of the infected roots. The sterile control showed patent dentinal tubules without bacteria on the root canal wall (Figure 1A). However, bacterial clumps and their extracellular matrix were observed in infected samples (Figure 1B and C). Furthermore, some dentinal tubules were invaded by bacteria (Figure 1D).

**Figure 2:**
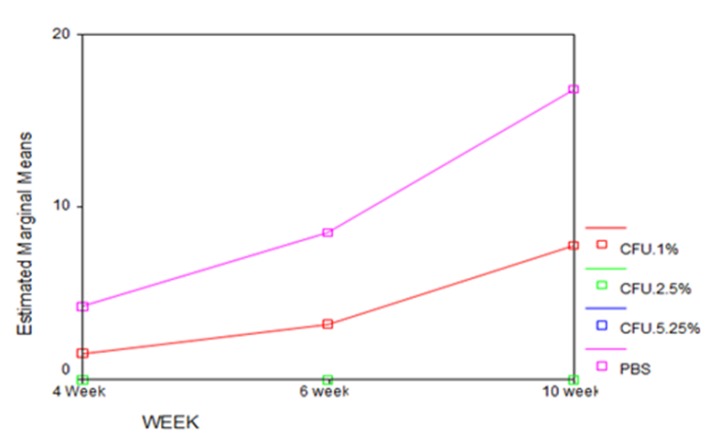
Growth of *E. faecalis* at 1%, 2.5%, 5.25% concentrations of NaOCl and PBS at weeks 4, 6, and 10. CFU: Colony formation unit.

The mean, mode, standard deviation, standard error, minimum, and maximum bacterial plate counts (CFU/mL) in each experimental group are shown in Table 1. As indicated in this table, not only was the mean bacterial count of *E. faecalis* in the PBS group in periods of 4, 6, and 10 weeks above the average P<0.0001), but also its mean bacterial count of 1% NaOCl was higher than those of 2.5% and 5.25% NaOCl (P<0.0001). No bacteria were observed in 2.5% NaOCl and 5.25% NaOCl groups (Table 1 and Figure 2). Also, multiple variables were compared to evaluate the significance of the differences between the groups (Table 2).

**Table 1:** Descriptive statistics of colony forming units (CFU) in terms of antibacterial treatment at different incubation times

**Type of detergent**	**Time **	**Mean**	**Mode **	**Standard Deviation (SD)**	**Standard Error (SE)**	**Minimum (Min)**	**Maximum (Max)**
1% NaOCl	Week 4	3.38	3	3.11	1.10	0	8
	Week 6	10.63	10.50	3.37	1.19	6	16
	Week 10	62.38	64.50	17.50	6.11	42	88
2.5% NaOCl	Week 4	0	0	0	0	0	0
	Week 6	0	0	0	0	0	0
	Week 10	0	0	0	0	0	0
5.25% NaOCl	Week 4	0	0	0	0	0	0
	Week 6	0	0	0	0	0	0
	Week 10	0	0	0	0	0	0
PBS	Week 4	18.38	17.50	4.92	1.74	11	26
	Week 6	73.38	74.5	14.56	5.14	48	91
	Week 10	292.88	292.50	107.21	37.87	104	416

SD: Standard Deviation, SE: Standard Error, Min: Minimum, Max: Maximum

**Table 2:** Post hoc Tukey analysis from one-way ANOVA shows the mean differences, P-values and 95% confidence intervals of CFU/mL data between each pair of experimental groups

** Major group **	** Minor group **	** Mean differences (A-B) **	**SE **	**Sig.**	** 95% Confidence interval **		** Minor group **	** Mean differences (A-B) **	**SE**	** Sig. **	** 95% Confidence interval **	
					** Lower **	**Upper **					**Lower**	**Upper**
*A*	*D *	2.73*	0.56	0.000	4.64	0.825	*I *	6.31*	0.56	0.000	8.22	4.40
	*H*	7.03*	0.56	0.000	8.95	5.12	*L *	15.29*	0.56	0.000	17.20	13.38
* B*	*D*	4.25*	0.56	0.000	6.16	2.34	*I *	7.82*	0.56	0.000	9.74	5.91
	*E*	3.22*	0.56	0.000	5.13	1.31	*L *	16.81*	0.56	0.000	18.72	14.90
	* H*	8.55*	0.56	0.000	10.46	6.64
*C*
	*D*	4.25*	0.56	0.000	6.16	2.34	*I *	7.82*	0.56	0.000	9.74	5.91
	* E*	3.22*	0.56	0.000	5.13	1.31	*L *	16.81*	0.56	0.000	18.72	14.90
	* H*	8.55*	0.56	0.000	10.46	6.64
* D *	*F*	4.25*	0.56	0.000	2.34	6.16	*J *	4.25*	0.56	0.000	2.34	6.16
	*G*	4.25*	0.56	0.000	2.34	6.16	*K*	4.25*	0.56	0.000	2.34	6.16
	*H*	4.30*	0.56	0.000	6.21	2.39	*L *	12.56*	0.56	0.000	14.47	10.64
	*I*	3.57*	0.56	0.000	5.48	1.66
* E *	*G*	3.22*	0.56	0.000	1.31	5.13	*J *	3.22*	0.56	0.000	1.31	5.13
	*H*	5.33*	0.56	0.000	7.24	3.42	*K*	3.22*	0.56	0.000	1.31	5.13
	*I*	4.60*	0.56	0.000	6.51	2.69	*L *	13.56*	0.56	0.000	15.50	11.67
*F*	*E *	3.22*	0.56	0.000	5.13	1.31	*I *	7.82*	0.56	0.000	9.74	5.91
	* H*	8.55*	0.56	0.000	10.46	6.64	*L *	16.81*	0.56	0.000	18.72	14.90
*G*	*H*	8.55*	0.56	0.000	10.64	6.64	*L *	16.81*	0.56	0.000	18.72	14.90
	*I*	7.82*	0.56	0.000	9.74	5.91
*H*	*J*	8.55*	0.56	0.000	6.64	10.46	*L *	8.25*	0.56	0.000	10.16	6.34
	* L*	8.55*	0.56	0.000	6.64	10.46
*I*	*J *	7.82*	0.56	0.000	5.91	9.74	*L *	8.98*	0.56	0.000	10.89	7.07
	*K*	7.82*	0.56	0.000	5.91	9.74						
*J*	*L *	16.81*	0.56	0.000	18.27	14.90						
*K*	* L *	16.81*	0.56	0.000	17.27	14.90						

A. 1% Sodium hypochlorite (NaOCl) at week 4; B. 2.5% NaOCl at week 4; C. 5.25% NaOCl at week 4; D. Phosphate buffer saline (PBS) at week 4; E. 1% NaOCl at week 6; F. 2.5% NaOCl at week 6; G. 5.25% NaOCl at week 6; H. PBS at week 6; I. 1% NaOCl at week 10; J. 2.5% NaOCl at week 10; K. 5.25% NaOCl at week 10; L. PBS at week 10.

## Discussion

The main objective of endodontic therapy is to prevent apical periodontitis. The bacteria grown in infected root canals play a crucial role in the develop-ment of apical periodontitis. Accordingly, decreasing or preferably eliminating the bacterial populations in the root canals can lead to an improvement in endodontic therapy.^14^ *E. faecalis* is the most common microorganism isolated from apical periodontitis after treatment. This anaerobic gram-positive coccus has several virulence factors that make it resistant to antimicrobial agents. Among these factors are the ability to endure food restrictions and poor diet for long periods of time, adhesion to the canal walls, invasion into the dentinal tubules, changing the host defenses, having lytic enzymes, resistance to common medications, and the ability to form solid biofilms.^15,16^ Bacterial biofilm is formed from a polymeric matrix that encloses bacterial populations in itself as well as from internal network canals which exchange and circulate food throughout the biofilm.^3^ Destruction of bacterial cells in a biofilm is nearly 1000 times more difficult than the bacterial cells in a planktonic structure.^5^ Shen et al^17^ showed that as bacterial biofilms age, various changes occur in their structure, which affect their physiological and metabolic function. Kishen et al^10^ demonstrated that after 4 weeks of incubation of *E. faecalis*, bacterial cells completely cover the dentin surface and after 6 weeks, mature biofilms which have a very coherent structure together with symptoms of mineralization, are formed. Previous studies also revealed that the resistance of *E. faecalis* biofilm against antibacterial agents is affected by the increase in incubation time (biofilm age) and the physiological status of cells.^18^

The results of this study indicated that when using 1% NaOCl solution, bacterial count in 10-week-old biofilm is higher than that in 6-week-old biofilm and in 6-week-old biofilm it is higher than that in 4-week-old biofilm. These results show that by increasing the incubation time and formation of a mature biofilm, removal of bacteria from their organized, calcified and very coherent structure becomes more difficult. Moreover, mature biofilm provides a specific environment that supports bacterial metabolic activities and, thereby, protects bacteria against bactericidal detergents. After 6 weeks of incubation of bacteria, bands of carbonate and phosphate apatite gradually increase on the surface of the biofilm making it a “supersaturated structure”.19 In this experiment, 1% NaOCl resulted in a decrease in the number of bacteria in all the three stages of biofilm compared to the control PBS biofilm, while 2.5% and 5.25% NaOCl completely inhibited the growth of living cells in all the 3 stages of biofilm incubation (4, 6 and 10 week-old biofilms). According to previous studies, NaOCl has a notable bactericidal effect on *E. faecalis* biofilms.^20,21^ All the concentrations of NaOCl are effective against biofilms of *E. faecalis*, but they exert their best antimicrobial effect in different exposure times.^22^ Gomez et al^23^ showed that 2.5% NaOCl is capable of killing all bacteria within 10 minutes. Based on our findings, 2.5% and 5.25% NaOCl can completely destroy mature and old biofilms of *E. faecalis* at all the time intervals, while 1% NaOCl only partially decreases bacterial count as compared with PBS.

## Conclusion

It can be concluded that the effect of 1% NaOCl in destroying bacteria decreases as the biofilm matures. In addition, the antimicrobial effect of NaOCl depends on two factors: contact time of the solution with biofilms and concentration of the solution. In this study, NaOCl contact time in all the three groups (1%, 2.5% and 5.25% NaOCl) was similar, but the results showed that in 1% NaOCl, time is not a key factor for the elimination of all *E. faecalis*s bacteria. However, in groups with 2.5% and 5.25% NaOCl, the solution was able to remove all the bacteria at all the time intervals due to the higher concentration of sodium hypochlorite.

Regarding the metabolic and physiological changes in the structure of *E. faecalis* biofilm over time, further studies with intermittent and longer incubation times are needed in order to better simulate clinical conditions in root canal treatment.

## Acknowledgments

The financial support of the Tabriz University of Medical Sciences is acknowledged.

## Authors’ contributions

MFR contributed to concepts, design and data acquisition. YR assisted in the concepts, design, clinical studies and data acquisition. MRN contributed to manuscript preparation and data analysis. LR assisted in the literature search and manuscript preparation. MM contributed to the literature search and manuscript preparation. MHSB contributed to clinical studies and data acquisition. YM assisted in manuscript preparation and editing.

## Funding

The authors appreciate the financial support of Tabriz University of Medical Sciences.

## Competing interests 

The authors declare no competing interests with regards to the authorship and/or publication of this article.

## Ethics approval

The authors considered all the ethical and the humanity issues and performed according to the Helsinki Declaration of 1975, as revised in 2008. All the experiments on human subjects were approved by the Ethics Committee of Tabriz University of Medical Sciences. Detailed informed consent form was obtained from all of the participants included in the study.
